# Assessing White Matter Microstructure in Brain Regions with Different Myelin Architecture Using MRI

**DOI:** 10.1371/journal.pone.0167274

**Published:** 2016-11-29

**Authors:** Samuel Groeschel, Gisela E. Hagberg, Thomas Schultz, Dávid Z. Balla, Uwe Klose, Till-Karsten Hauser, Thomas Nägele, Oliver Bieri, Thomas Prasloski, Alex L. MacKay, Ingeborg Krägeloh-Mann, Klaus Scheffler

**Affiliations:** 1 University Children's Hospital Tübingen, Germany; 2 High Field Magnetic Resonance, Max-Planck Institute for Biological Cybernetics, Tübingen, Germany; 3 Biomedical Magnetic Resonance, University Hospital Tübingen, Germany; 4 Institute of Computer Science, University of Bonn, Germany; 5 Department Physiology of Cognitive Processes, Max Planck Institute for Biological Cybernetics, Tübingen, Germany; 6 Department of Diagnostic and Interventional Neuroradiology, University Hospital, Tübingen, Germany; 7 Radiological Physics, University of Basel, Basel, Switzerland; 8 University of British Columbia, Vancouver, Canada; University of Minnesota, UNITED STATES

## Abstract

**Objective:**

We investigate how known differences in myelin architecture between regions along the cortico-spinal tract and frontal white matter (WM) in 19 healthy adolescents are reflected in several quantitative MRI parameters that have been proposed to non-invasively probe WM microstructure. In a clinically feasible scan time, both conventional imaging sequences as well as microstructural MRI parameters were assessed in order to quantitatively characterise WM regions that are known to differ in the thickness of their myelin sheaths, and in the presence of crossing or parallel fibre organisation.

**Results:**

We found that diffusion imaging, MR spectroscopy (MRS), myelin water fraction (MWF), Magnetization Transfer Imaging, and Quantitative Susceptibility Mapping were myelin-sensitive in different ways, giving complementary information for characterising WM microstructure with different underlying fibre architecture. From the diffusion parameters, neurite density (NODDI) was found to be more sensitive than fractional anisotropy (FA), underlining the limitation of FA in WM crossing fibre regions. In terms of sensitivity to different myelin content, we found that MWF, the mean diffusivity and chemical-shift imaging based MRS yielded the best discrimination between areas.

**Conclusion:**

Multimodal assessment of WM microstructure was possible within clinically feasible scan times using a broad combination of quantitative microstructural MRI sequences. By assessing new microstructural WM parameters we were able to provide normative data and discuss their interpretation in regions with different myelin architecture, as well as their possible application as biomarker for WM disorders.

## Introduction

The assessment of white matter (WM) microstructure generally aims at detecting, characterising and quantifying suspected myelin pathology. These changes in the myelination of WM pathways have been shown to directly affect brain connectivity resulting in disease conditions and behavioural variation [1,2]. In a clinical context, quantitative information about WM microstructure can not only help in identifying the aetiology of a disease but can also provide biomarkers for evaluating the dynamics of the disease course and/or for evaluating treatment effects [3–5].

Advances in magnetic resonance imaging (MRI) techniques make the quantification of putative WM biomarkers increasingly robust and standardised, thus enabling direct comparisons between different scanners and study sites [6–8]. Moreover, it is likely that such advances will enable the characterisation of histology-like features of the white matter in the near future [9–12].

There are several established MR methods available that are sensitive to different aspects of WM microstructure, and hence of interest for the study of WM pathology. Most methods are based on the notion that magnetic properties of water surrounded by densely packed myelin are profoundly different from water moving and exchanging more freely inside the cytosol of neuronal and glial cell bodies or water in the extra-cellular space. MR-derived parameters such as the longitudinal (T1) and (effective) transverse (T2*) T2 relaxation times, the proton density, the magnetic susceptibility, the magnetization transfer (MT) effect, as well as different measures related to water diffusion are sensitive to microstructure at sub-voxel length scales. Great care was taken to select the best candidate methods that are sensitive to different aspects of the microstructure, yet can be combined in a measurement protocol of limited total duration for future use in our Children’s hospital.

A fundamental MR parameter is the longitudinal relaxation time, T1, which determines the time that a perturbed nuclear spin distribution needs to get back to equilibrium. It was shown early that in brain tissue this process is presumably driven by the lipids in the myelin sheaths [13].A more recent study demonstrated that variations in brain T1-maps can be predicted by a linear combination of two other MR parameters, magnetization transfer and effective transverse relaxation [14]. Indeed, the main contributor to T1-contrastin the brain seems to be the chemical exchange of bound and free water pools, which was parameterised by exploiting the magnetization transfer (MT) effect, and we therefore, chose to directly assess this MR parameter, rather than measure T1 in white matter tracts.

In Magnetization Transfer (MT) imaging, the effect of saturating the signal from extremely short-lived water pools, for instance originating in the lipid bilayer of myelin, is indirectly observed by comparing the MR-visible water with and without saturation transfer pulses applied on the bound pool. Indeed, the bound water pool has a very short T2 and therefore gives rise to a resonance line which is much broader than the MR-visible resonance and can therefore easily be targeted by saturation RF pulses [15]. For a full quantification of this biophysical effect, complex modelling of different water pools and quantification of the different T1 times of the pools is necessary. This endeavour can be facilitated by the dynamic analysis of increasing levels of saturation achieved by repetitive MT-pulses [16]. As an alternative to a full quantification of the multi-compartmental processes of MT, the assessment of the magnetization transfer ratio (MTR) has been shown to be clinically useful, e.g. in WM disorders [4,17]. In the present study, the fast bSSFP MT method is used, which has been shown to be a robust, sensitive and very fast measure of MTR [7,18,19].

Other methods capable of directly capturing the MR signal originating in water pools trapped between myelin sheaths are based on the transverse relaxation time, T2. The indirect effect of myelin on these water pools provokes a loss in MR-signal coherence, which is more rapid than for water pools located within the axons or in the cytoplasm. Measurements of the T2 difference between pools have proven highly relevant for characterising white matter. Pioneered by the group of MacKay [20], the separation of multiple water components by T2 relaxometry is a relatively straightforward and well-established measure of myelin water that has been shown to correlate well with histology and to remain unaffected by inflammation or unspecific changes in the overall water content [21]. Basically, by applying a train of 180° refocusing pulses (the so-called Carr-Purcell-Meibom-Gill experiment or CPMG, after its inventors) the method separates components with a long T2 time arising from the mobile water pool and the myelin trapped water with short T2 times, being below 30ms at 3T. A few caveats that have been dealt with over the years relate to the presence of stimulated echoes arising due to imperfect 180° pulses across the slice thickness and magnetization transfer effects arising from the excitation of spins in adjacent slices in case of multi-slice 2D imaging. The solution to this has been to take into account the presence of stimulated echoes in the modelling of the MR signal decay [22], on one hand, and to perform single-slice measurements with increased bandwidth for the refocusing pulses, on the other hand. These improvements have rendered the method more robust. In our study, we used the 2D CPMG approach in order to focus our investigation on a single slice with the same spatial coverage as the MR spectroscopy sequence.

Proton MR spectroscopy (MRS) offers an insight into the brain chemistry and therefore information about the composition of certain cellular components. However, the metabolic spectrum of myelin is complex with many overlapping functional groups [23] as well as compounds with short T2-relaxation times that hampers their detection [24]. Still, certain metabolite peaks have been found to be enriched in neurons or myelin membranes. Therefore, MRS gives additional valuable information for the characterisation of WM, although the technique most likely is inadequate for directly quantifying myelination [4]. The metabolite N-acetylaspartate (NAA) has been described to be enriched in neuronal structures [25,26], whereas Choline-containing compounds (Cho) were found to be associated with myelin membranes [27,28]. These metabolites show some regional variation throughout the brain, probably due to the different underlying tissue composition [29,30].

A MR parameter which is highly sensitive to the presence of myelin is the magnetic susceptibility, which basically is a measure of how well the static magnetic field can penetrate the tissue. In tissues that are more paramagnetic than the surrounding tissue, the local field experienced will be slightly higher, while the reverse happens in more diamagnetic areas. The phase of the MR image can be seen as a ‘finger print’ of the local magnetic field distribution, and regional variations in susceptibility can thus be probed through this parameter yielding higher phase values in paramagnetic areas (although the definition may differ between scanners, [31]). The white and grey matter show a strong difference in magnetic susceptibility leading to contrast differences in phase images, first observed at 7T [32]. To overcome limits posed by the orientation dependence and non-local components of the phase effect, methods that allow conversion of the phase maps into quantitative susceptibility maps (QSM) have been developed [33,34]. QSM gives a measure of the average magnetic susceptibility distribution in every unit volume (voxel)and, hence, can be used to map regional changes in tissue microstructure [35]. For clinical use, QSM is an emerging method, and for this reason we included it in a small cohort of subjects in our study. Since a multi-echo gradient echo sequence was used for this purpose, we also evaluated the presence of R2* effects.

Diffusion-weighted imaging (DWI)sequences are sensitized to detect the motion of water molecules, and hence tissue properties, in the range of micrometers [36,37]. The most commonly applied model in order to quantify diffusion in the brain is the tensor model [38], forming the basis of diffusion tensor imaging (DTI). By estimating the eigen-values from this tensor, different measures that characterises diffusion along (= axial diffusivity) and perpendicular (= radial diffusivity) to the fibre pathways can be assessed. More commonly, all eigen-values are combined into the dimensionless metric fractional anisotropy (FA);while the trace of the tensor is proportional to the mean diffusivity (MD) per voxel [39,40]. While DTI metrics are useful for characterizing coherent fibre bundles, the technique is of limited use for WM areas containing crossing fibres [39,41–44]. As the diffusion tensor can only detect one major diffusion direction per voxel, radial and axial diffusivities can be interpreted as diffusivities across and along the bundle only in voxels with one (parallel) fibre population, which has been shown to be true only for 10% of WM voxels [45], limiting the interpretation of DTI-parameter considerably [41]. As a valuable extension of DTI, diffusion kurtosis imaging (DKI) has been introduced [46]. By measuring the non-gaussian part of the diffusion signal, it increases the sensitivity to pathological changes [47]. Still, besides the lack of specificity to cellular components [48], DKI is also affected by the problem of crossing fibres, where the axial and radial kurtosis is difficult to interpret [49]. To overcome this major limitation a scan protocol that includes multiple diffusion-weightings can be used to derive additional DWI parameters separating the highly restricted water diffusion inside the axon from the less hindered diffusion in the extra-axonal space [50]. Such approaches have been shown to reveal the underlying fibre architecture and provide microstructural parameters with improved sensitivity to pathological changes and better specificity to histological tissue properties than the more classical DTI-based metrics [51–53]. While it has been argued that a strict compartmentalization of the tissue might be an oversimplification of modelling the diffusion signal [54], several promising models have been introduced and validated by histology [9,10,55,56], although some of them require very long scan times. One of the recently introduced methods that can be achieved within clinically feasible scan times, is neurite orientation dispersion and density imaging (NODDI) [57]. Although used with certain assumptions, it attempts to disentangle and quantify intra-axonal water diffusion and fibre dispersion, making it valuable for the purpose of our study.

**Fig 1:**
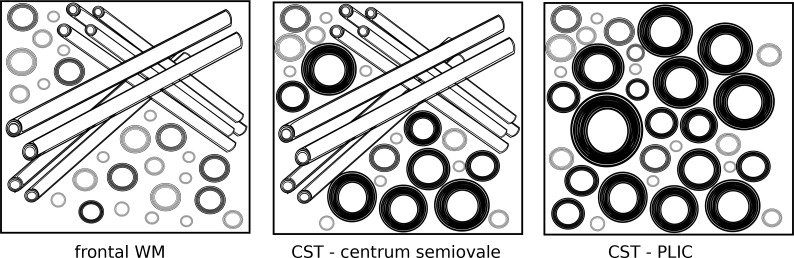
Illustration of different underlying fibre architecture in the three measurement regions as found by histology (e.g. [58–61]: Cortico-spinal tract (CST) fibres include those with larger axon diameter and thicker myelin sheaths, which show more parallel arrangements at the level of the posterior limb of internal capsule (PLIC) and more crossing fibres at the level of the centrum semiovale (CS). On the other hand, the frontal white matter (WM) region also contains crossing fibres, however, with thinner myelin sheaths and lesser axon diameter.

White matter microstructure is known to vary across the brain. Especially fibres of the cortico-spinal tract (CST) are known to have different tissue properties, compared to e.g. more frontal WM pathways, containing larger pyramidal fibres with increased axonal diameter and larger myelin sheaths [58]. Fig 1 illustrates the expected underlying differences in fibre architecture in three selected WM regions, according to histological data [58–61]. In the posterior limb of the internal capsule (PLIC), tightly packed and highly myelinated parallel fibre bundles of the CST with larger axon diameters run through and proceed towards the primary motor area. They pass the level of the centrum semiovale (CS), where these large-diameter axons are still highly myelinated, but run through WM regions with crossing fibres from commissural (corpus callosum) and association pathways (superior longitudinal fasciculus) [62]. On the other hand, the frontal WM region is characterised by a mixture of crossing fibres that interconnect between neocortical areas and connect to other brain areas further downstream, showing thinner myelin sheaths and smaller axon diameters as well as larger extracellular spaces (Fig 1).

Such microstructural differences have been shown to be detectable bya series of different MRI sequences [63–65]. Furthermore, even along the CST, the regional variation in fibre architecture (crossing/bending/single fibre populations) influence microstructural MRI parameters, resulting e.g. in different DTI-derived values at the level of the internal capsule and the centrum semiovale [62,65].

These areas were thus considered appropriate to probe WM microstructure in regions with different underlying fibre architecture, with our combined set of techniques. Due to its functional relevance and known unique microstructural features [58,63–65], the CST was assessed both at the level of the PLIC and the centrum semiovale and compared to a WM region in the frontal lobe for the whole brain techniques. The described MRI parameters were thus investigated, probing their ability to detect the known differences in microstructure in the selected regions. Furthermore, we aimed to provide and evaluate this myelin-sensitive MRI protocol to non-invasively probe WM microstructure in a clinical context, thereby providing normative data and interpretation of new microstructural WM parameters aimed for studies of juvenile and adult forms of leukodystrophies [66]. Therefore a matching age range was selected, covering the adolescence and young adulthood.

## Methods

### Subjects

Nineteen typically developing healthy adolescents (median age 14.9 years, range 9–32 years, 10 females) were recruited as controls for an ongoing study about juvenile leukodystrophies, covering the age range of the expected patients. The age distribution between the two genders were kept similar with males having a median age of 14.9 years (range 9–32 years) and females having a median age of 14.8 years (range of 10–24 years), therefore reducing the impact of a gender bias for this study. A standardised gross motor function test was performed in all subjects (GMFM 88) and demonstrated normal gross motor function in all subjects (100%). In addition, the Wechsler Intelligence Scale for children or adults was performed. The total IQ values in this group ranged from 103 to 130 (mean 114.6, SD 6.89). The study was approved by the local ethics committee. All volunteers and/or their legal guardians gave their written informed consent for participation in the study.

### MRI acquisition

An MRI sequence protocol was established on a 3Tesla wide-bore scanner (“Skyra”, Siemens Healthcare GmbH, Erlangen, Germany) equipped with a 2-channel body transmit coil and a 32-channel head receiver coil, to allow data acquisition within a total (net) examination time of 42 minutes. Conventional MRI sequences consisted of 4 acquisitions, two T2-weighted turbo-spin echo scans acquired with axial (40 slices, acquisition time (TA) = 1:59min) and sagittal (30 slices, TA = 1:59min) image orientations (with the following sequence parameters: repetition time(TR)/echo time (TE) = 10810/84ms, field of view (FoV) = 190x192mm^2^, matrix size = 384x384, slice thickness = 3mm, reconstructed to a voxel size of 0.5x0.5x3mm^3^, 2 averages, grappa factor of 2); an axial T2-weighted fluid attenuated inversion recovery (FLAIR) sequence (TA = 1:30min, TR/TE = 8800/85ms, inversion time (TI) = 2480ms, flip angle = 150°, FoV = 172×230 mm^2^, matrix = 134×256, slice thickness 3mm, 40 slices, voxel-size: 0.9x0.9x3mm^3^, grappa factor of 3); and a T1-weighted Magnetization-Prepared Rapid Acquisition Gradient Echo (MPRAGE) sequence (TA 3:27min, TE/TI/TR = 4.11/900/2300ms;FA = 9°, FoV = 256×256 mm^2^,matrix size 256×256, 176 slices, voxel-size = 1 mm isotropic, grappa factor of 3). All axial images were acquired in the AC-PC orientation.

In addition to the conventional sequences, we also acquired data for the processing of several quantitative parameter maps. The diffusion-weighted (DW) images were acquired using a high angular resolution twice-refocused echo planar imaging (EPI) read-out with two shells (TA = 15:10min, 64 directions with b = 2000s/mm^2^; 30 directions with b = 700s/mm^2^, TE/TR = 89/9100ms, FOV = 192×192mm^2^; 96×96 matrix, slice thickness = 2mm, 50 interleaved contiguous slices, voxel size = 2×2×2mm³, bandwidth 1796 Hz/Px, grappa factor of 2). For EPI distortion correction, an additional b = 0 image (TA = 0:09min) was acquired with the same resolution, FoV and readout bandwidth, but with reversal of the direction of acquisition along the phase-encode (PE) axis [67].

For MR spectroscopy, two approaches were used: a chemical-shift imaging (CSI) and a single voxel (SV) sequence in order to evaluate the sensitivity and robustness of the sequences. In nine subjects, a CSI sequence with the same axial orientation as the axial T2-weighted image positioned above the lateral ventricles(TA = 3:57min, TR/TE = 1600/135ms, FoV = 160x160mm^2^; matrix size 12x12, interpolated to 32x32; slice thickness = 12mm, voxel size 5x5x15mm³). In another ten subjects, a SV PRESS-sequence was performed in two locations within the level of the CSI slice: one in the left frontal WM and one in the parietal WM at the central region using the central sulcus as a landmark (where the CST was expected to be located) (TA = 1:49min, TR/TE: 1600/135ms, 64 averages, voxel size 15x15x15mm^3^, bandwidth 1000 Hz, vector size 1024, 4 preparation scans, water suppression with bandwidth 35Hz, flip angle 90°).

Using the same localization as the CSI sequence, a Carr-Purcell-Meiboom-Gill (CPMG) sequence (TA = 4:17min, TR = 3s, TE = 10ms to 320ms in steps of 10ms, FoV = 192x192mm^2^; matrix size 128x128, slice thickness = 5mm, 1 slice, voxel size = 0.75×0.75×5mm^3^) was acquired, modified to have shorter RF pulse durations and increased bandwidth of the 180° RF pulse than the standard vendor sequence. B1-mapping for each transmit channel was performed and combined using the manufacturer’s standard approach and a voxel size that matched the CPMG sequence (TA = 0:10min, TR/TE 5000/1.72ms, nominal flip angle 8°, FoV = 192x192mm^2^; matrix size 128x128, slice thickness = 5mm, 1 slice, voxel size = 0.75×0.75×5mm^3^).

Two sets of balanced steady state free precession(balanced SSFP) 3D images were acquired using slab selective RF pulses (flip angle of 20°, FOV = 256x256mm^2^, matrix size = 192×192, 144 slices, isotropic voxel size1.3mm^3^, reconstructed to0.65x0.65x1.3mm³). One 3D data set was acquired with a long duration of the excitation pulse:1500μs (TA = 3:05min, TE/TR = 2.12/4.23ms) to minimize Magnetization Transfer (MT) effects while the second data set had a RF-pulse duration of200μs (TE/TR = 1.47/2.93ms) for the MT-weighted scans.

Images for quantitative susceptibility mapping (QSM) and R2* mapping were acquired with a 3D flow-compensated gradient-echo sequence at eight (4.5/10/15/21/27/33/39/46ms) different echo times using a TR of 50ms, flip angle of 15° and a voxel size of 1x1x2mm^3^ (N = 5).

### Image processing

Motion and eddy-current correction was applied to the DWI data set as described before [68]. In addition, correction for EPI distortions was performed for improved alignment with anatomical images prior to calculation of the diffusion tensor [69,70]. The data was corrected for variation in signal intensity during scanning using the b0 images [71]. Maps of mean diffusivity (MD), fractional anisotropy (FA), as well as parallel (or axial, λ_1_) and perpendicular (or radial, λ_(2+3)*0.5_) eigenvalues were calculated using the standard log-linear least squares fit of the tensor model [38,72]. For the calculation of the DTI parameters, only the low b-value shell was used (30 directions with b = 700s/mm^2^) for better comparability to “classic” DTI studies.

The diffusional kurtosis model was fitted using quadratic cone programming. Similar to Tabesh et al. [73], we performed a linear least squares fit with constraints on diffusivities and kurtosis values that improve robustness to noise. Our implementation is based on the cvxopt package (cvxopt.org) and imposes a semi-definiteness constraint on diffusivities (d≥0.1 μm^2^/ms), since lower values are implausible and would render Tabesh’s upper bound on kurtosis ineffective. Maps of mean kurtosis (MK), radial kurtosis (RK), and axial kurtosis (AK) were computed based on the equations provided by Tabesh et al. [73].

Furthermore, NODDI-derived parameters, including intracellular volume fraction (ICVF, neurite density), orientation dispersion (ODI), and free water fraction (ISO) were calculated based on the publicly available implementation of the NODDI three—compartment model [57]. Even though the ICVF parameter has been referred to as a “measurable water fraction” rather than a volume fraction, as a reminder of the fact that water inside the myelin does not contribute to the DWI signal [52], we will keep the more widely used terminology.

MRI Spectra were analysed quantitatively using the LCModel [74]. Absolute concentrations of N-acetylaspartate and N-acetylaspartylglutamate (NAA), creatine and phosphocreatine (Cr), as well as choline containing compounds (Cho) were calculated. For this study, only ratios of NAA/Cr and Cho/Cr were further analysed.

MWF was fitted voxel-wise [22], in 2 steps. First, all parameters including the flip angle were fitted. In the second step, the flip angle was fixed to the voxel-specific value measured by the B1 scaled to match the median value found in the first step. Number of fitted T2 points: 32; Chi^2^ regularization of 1.01.

The MT ratio (MTR) map was calculated in percentage units from the co-registered non-MT and MT images using the following equation: (non-MT–MT) / non-MT.

For QSM the frequency maps were generated by using the phase unwrapping and multi-echo fitting tool from the MEDI-Toolbox [75] and the BET masking tool from FSL [76]. Background dipole field modulations were removed with RESHARP [77] and the local dipole inversion process was performed by using the fast magnitude-weighted L1-regularization technique with total variation penalty [78]. No rescaling of the QSM values was undertaken.R2* mapping was performed by assuming a mono-exponential signal decay. Non-linear least square fitting based on the Levenberg-Marquard algorithm was made pixel-wise for the square of the MRI signal decay, to minimize the influence of pixels and time-points with low signal.

For each subject, conventional images, MWF, MTR, QSM, and R2*were all co-registered (rigid body transformation using normalized mutual information) and interpolated to the subject’s FA map, therefore it was made sure that all image modalities per subject were transformed into the same space and image dimensions for further analysis.Images were processed and analysed using MRtrix (version 0.3.12, www.github.com/MRtrix3/mrtrix3 [79]), Matlab (version R2014b, www.mathworks.com) and FSL tools (version 5, http://fsl.fmrib.ox.ac.uk/fsl/fslwiki/FSL, [80]).

### Data analysis

**Fig 2:**
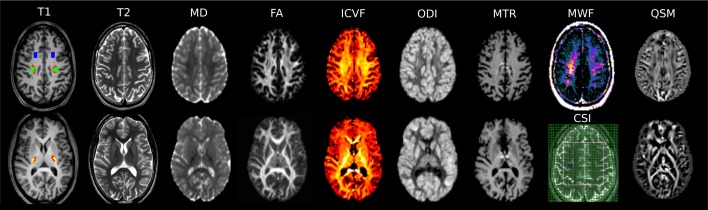
MRI protocol for this study. T1-weighted MPRAGE image (T1, left) in two different axial planes of a 17y-old typically developing subject indicating the measurement ROIs guided by the cortico-spinal tract fibre s outlined in red: frontal WM (in blue), cortico-spinal tract at the level of the centrum semiovale (in green) and at the level of the posterior limb of the internal capsule (in yellow). Other image parameters included in the protocol were T2-weighted axial TSE (T2), mean diffusivity (MD), fractional anisotropy (FA), intracellular volume fraction (ICVF), orientation dispersion (ODI), Magnetization Transfer Ratio (MTR), Myelin Water Fraction (MWF), Chemical Shift Imaging (CSI)–MR Spectroscopy, and quantitative susceptibility mapping (QSM, the white matter fibers have been highlighted by rendering diamagnetic effects bright, and paramagnetic dark).

For data analysis, a region of interest (ROI) approach was chosen focusing on the three regions illustrated in Fig 1:Two regions along the cortico-spinal tract (CST) were selected, one at the level of the internal capsule, CST-PLIC, and another one located in the centrum semiovale, CST-CS, at the level where the CSI slice was placed, just above the ventricles (see Fig 2). The CST was tracked in each individual using manual delineation of the pre-central gyrus and the descending pathways at the level of the ponsas a seed as described previously [62]. Probabilistic tractography was performed using constrained spherical deconvolution using MRtrix [79,81]. A third ROI was defined in the frontal WM at the level of the MWF and CSI slice, anterior to the CST ROI (Fig 2). In order to avoid any bias of the ROI definition related to the local MR signal intensity in the modalities of interest, ROIs were drawn onto the co-registered conventional T1-weighted images with the CST overlaid, where signal intensity throughout the WM is homogeneous.

For each ROI mean values were calculated across the two hemispheres, since the objective of this study was to compare between ROIs, and not to explore the hemispheric lateralization of the parameters. Boxplots of quantitative image parameters were displayed and (paired t-test) statistical analysis was conducted by paired t-test comparing the results between the ROI locations. As this study with a relatively small sample size was considered exploratory, the p-values for these analyses were not corrected for multiple comparisons and can therefore be regarded as being descriptive.

## Results

**Fig 3:**
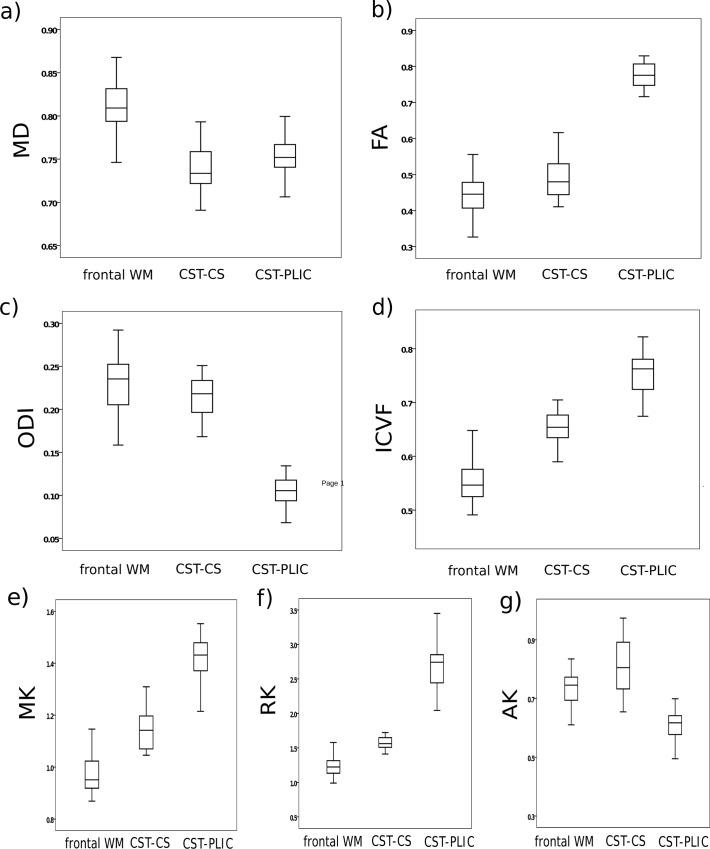
**Boxplots of diffusion-weighted imaging parameters**(Units of diffusivities are given in μm^2^/ms) included **a)** mean diffusivity (MD), **b)** fractional anisotropy (FA), **c)** orientation dispersion (ODI), **d)** intracellular volume fraction (ICVF),**e)** mean kurtosis (MK), **f)** radial kurtosis (RK), and **g)** axial kurtosis (AK). Parameters were measured in frontal white matter (WM) and two parameters within the cortico-spinal tract (CST): at the level of the posterior limb of internal capsule (PLIC) and at the level of the centrum semiovale (CS), see also Fig 1.

**Fig 4:**
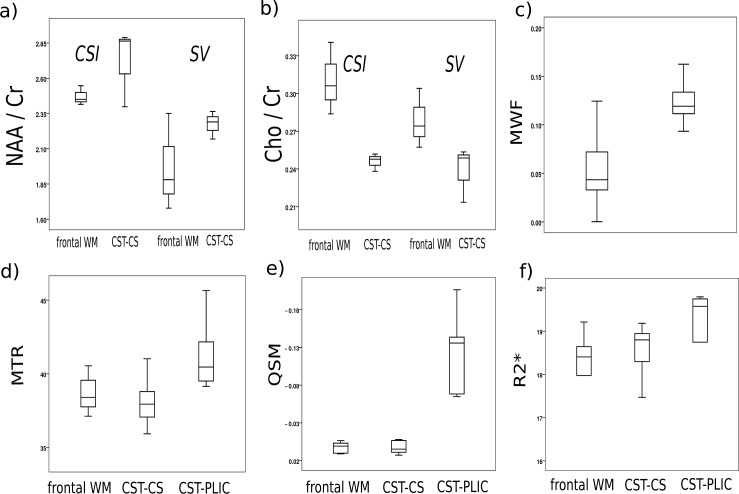
**Boxplots of quantitative parameters** included **a)** ratio of N-acetylaspartate and N-acetylaspartylglutamate (NAA) to creatine and phosphocreatine (Cr) both for chemical shift imaging (CSI) and single voxel (SV) measurements, **b)** ratio of choline containing compounds (Cho) to Cr both for CSI and SV, **c)** myelin water fraction (MWF), **d)** magnetization transfer ratio (MTR), **e)** quantitative susceptibility mapping (QSM), and **f)** R2*. Parameters were measured in frontal white matter (WM) and two parameters within the cortico-spinal tract (CST): at the level of the posterior limb of internal capsule (PLIC) and at the level of the centrum semiovale (CS), see also Fig 1.

Fig 2 shows the MRI results obtained by different modalities from one 17 year-old male subject. In Table 1, the results for the ROI measurements are listed and reproduced in boxplots (Figs 3 and 4). Most microstructural MRI parameter showed significant differences between the investigated WM regions.

**Table 1:** Measurement results in the three regions of interest: the frontal WM, the CST at the level of the centrum semiovale (CST-CS), and at the level of the posterior limb of the internal capsule (CST-PLIC).

Parameter	Frontal WM		CST–CS		CST–PLIC		T-score(paired t-test)	
	mean	SD	mean	SD	mean	SD	frontal vs CST-CS	CST-CS vs PLIC
MD	0.810	0.032	0.740	0.028	0.752	0.025	10.3***	-1.81
FA	0.440	0.061	0.488	0.057	0.778	0.035	-3.03**	-19.2***
λ_1_	1.207	0.078	1.179	0.089	1.611	0.082	1.44	-17.4***
λ_(2+3)*0.5_	0.611	0.046	0.523	0.035	0.318	0.035	7.03***	17.8***
MK	0.972	0.074	1.144	0.075	1.392	0.132	-13.2***	-8.26***
RK	1.227	0.144	1.601	0.138	2.600	0.463	-12.0***	-9.36***
AK	0.732	0.063	0.814	0.091	0.604	0.056	-3.34**	7.33***
ICVF	0.551	0.038	0.656	0.038	0.745	0.067	-15.0***	-5.73***
ODI	0.230	0.036	0.215	0.027	0.104	0.019	1.53	15.4***
ISO	0.045	0.025	0.067	0.020	0.106	0.035	-4.38***	-4.94***
NAA/Cr (CSI)	2.422	0.188	2.828	0.270			-5.48***	
Cho/Cr (CSI)	0.278	0.034	0.238	0.019			4.03**	
NAA/Cr (SV)	2.266	0.365	2.556	0.420			-3.28*	
Cho/Cr (SV)	0.295	0.019	0.250	0.020			7.34***	
MWF	0.053	0.033	0.125	0.034			-12.0***	
MTR	38.91	1.842	38.08	1.519	40.94	1.803	3.06**	-10.09***
QSM	0.0024	0.0079	0.0023	0.0093	-0.1238	0.0589	0.025	4.28***
R2*	17.97	1.380	18.54	0.680	18.17	2.917	-1.575	0.355

Units of diffusivities are given in μm^2^/ms. Statistically significant differences are highlighted in bold script (*p<0.05, **p<0.01, ***p<0.001).

Diffusion imaging revealed higher MD in the frontal WM ROI than in the CST-CS (p<0.01) and CST-PLIC (p<0.01) ROIs without differences in MD between CST-CS and PLIC (see Fig 3A). DTI-derived parameters showed significantly higher parallel and lower radial eigenvalues in the CST-PLIC region, resulting in a higher FA (p<0.001) (Fig 3B). Similarly, smaller but still significant differences with higher radial (p<0.01)and higher parallel (p = 0.066) eigenvalues were found in the frontal WM than in the CST-CS regions, resulting in a lower FA in the frontal WM region compared to CST-CS (p = 0.003).

DKI parameters revealed highest values for MK and RK in the CST-PLIC (p<0.001), clearly lower values in CST-CS, and even lower ones in the frontal WM (p<0.001). Differences in AK between the ROIs were less pronounced but still significant with lowest AK in CST-PLIC and highest in CST-CS (p<0.001).

The NODDI parameters also showed significant regional differences. The neurite density (ICVF, Fig 3D), was highest in the CST-PLIC, clearly lower in the CST-CS (p<0.001) and lowest in the frontal WM (p<0.001). In contrast, the ODI parameter (Fig 3C) was similar in the frontal WM and the CST-CS, but was significantly lower in the CST-PLIC (p<0.001).

The MR Spectroscopy (Fig 4A and 4B) values revealed higher NAA/Cr ratios in the CST-CS compared to the frontal WM, regardless of acquisition methodology, with slightly lower standard deviations, and hence improved significance for the CSI approach (p<0.001) than for SV measurements (p = 0.011). On the other hand, both methods showed lower Cho/Cr ratios in the CST-CS region than in the frontal WM ROI (p<0.01).

Quantitative values obtained by Myelin water fraction imaging (MWF, Fig 4C) were found to be significantly higher in the CST-CS region compared to the frontal WM (p<0.001). The Magnetization Transfer Ration (MTR), however, was slightly higher in frontal WM compared to the CST-CS region, while significantly higher MTR values were found in the CST-PLIC than in the frontal WM and CST-CS region (p<0.001). Similarly, we found no significant differences between the CST-CS and the frontal WM in the QSMs while a clearly higher diamagnetic susceptibility was measured in the CST-PLIC (p<0.001).On the other hand, R2* was not significantly different between the three ROIs.

## Discussion

In this study we were able to probe WM microstructure by MRI in brain regions with different underlying fibre architecture. In a clinically feasible scan time, various myelin-sensitive quantitative MR methods could be acquired. The results demonstrate that the underlying (normal) WM fibre architecture influences significantly and differently the behaviour of several quantitative MRI parameters.

High values were observed in all myelin-sensitive MR parameters assessed along the CST, including MTR, QSM, MWF, FA, MK, and ICVF, reflecting the unique microstructural characteristics of the CST with thicker myelin sheaths and tightly packed and partly larger fibres (Fig 1) [58–61]. Aside with this finding the MD values were low, reflecting a high degree of diffusion restriction and little free extracellular water diffusion due to aligned and tightly packed (myelin) membranes. On the other hand, in the frontal WM region, we observed higher MD and Cho/Cr levels, and lower FA, NAA/Cr, ICVF, and MWF levels, when compared to the more parietal WM of the centrum semi-ovale, where the CST fibres pass through. Interestingly, we found no differences in ODI and QSM between these two areas, suggesting that these tissue parameters are less influenced by the different proportions of intra- to extra-cellular compartments. ODI and QSM are known to reflect microstructural directionality, which is similar in the two crossing fibre regions (Fig 1).

In the following we will more critically assess and interpret our findings for the different MR modalities.

### DWI parameters

The cortico-spinal tract is known to contain fibres with larger axon diameter and myelin thickness due to long descending fibres mainly from the primary motor cortex [58,82]. Since intra-axonal water diffusion is restricted mainly by cell membranes and myelin sheaths, a thicker myelin in the CST leads to a decrease of measured apparent diffusivities radial to the main fibre direction, and to an increase in Fractional Anisotropy. Accordingly, in the frontal WM region water diffusion was less hindered as indicated by a higher MD and a lower FA. Also, MK was highly increased in the PLIC-CST (mainly due to increased radial kurtosis), in line with previous reports [83] and the association of diffusion kurtosis with myelination [84,85]. At the same time, higher intra-axonal water diffusion along the CST leads to higher axial diffusivity, further contributing to the high FA in the CST-PLIC. Here, axial kurtosis was lower compared to crossing fibre regions of the centrum semiovale, similar to previous observations [47].

The microstructural differences between these areas were also evident from the ICVF parameter, in line with results shown recently [11]. Clearly higher ICVF values were found in both CST-regions, compared to the frontal WM, most likely reflecting the increased intra-axonal water diffusion of the larger axons in the CST. Although it has to be noted that ICVF alone might not sufficiently be able to disentangle the influences of axonal density and myelination [52].

When interpreting diffusion parameters, the dispersion of fibre orientations isknown to be a critical factor, limiting the interpretation of FA or eigenvalues as measures for fibre integrity in crossing fibre regions [43,44,86]. As a result, parallel fibre arrangements, indicated by a low ODI, like in the PLIC, resulted in high FA, based on low radial and high parallel diffusivity as modelled by the diffusion tensor. These results are in line with previous diffusion studies of the CST, which showed high anisotropy at the level of the PLIC and low anisotropy at the level of the centrum semiovale [62,64,65]. On the other hand, in voxels with more complex fibre orientations, like crossings and fanning in the frontal WM and centrum semiovale level of the CST, corresponding FA and eigenvalues are highly influenced by the inhomogeneity of fibre orientations, making them difficult to interpret [44,62]. The same holds true for the diffusion kurtosis measures. While the mean kurtosis has been reported to be sensitive to pathological WM changes, and correlated with myelination and maturation of WM [84,85], it is difficult to interpret axial and radial kurtosis in regions of crossing fibres [49].

We found that the ICVF parameter was less influenced by the spread of fibre orientation, better reflecting the integrity of WM pathways in crossing fibre regions, as was recently shown by others and underlined by histology [10]. Interestingly, a recent study also showed ICVF to be more sensitive than FA in detecting brain maturation and myelination [87,88]. The same has been found in a comprehensive recent study [41], where a more specific axonal density parameter was found to be highly correlated with myelin content, whether in single or crossing fibre regions, different to tensor parameters (like FA), which were limited by the effect of crossing fibres.

While the assessment of conventional DTI parameters requires less scan time, the use of a high angular resolution sequence with different b-values allows a more detailed analysis of the diffusion signal per voxel [39]. Both crossing fibres and different diffusion compartments can be investigated by adding only a few more minutes of scan time. The additional data enables the calculation of other microstructural parameter maps, like in our study. Other recently developed and promising DWI parameters include apparent fibre density [89], hindrance modulated orientational anisotropy [90],axon diameter [91,92], neurite density and dispersion [57,93], permeability [94], tortuosity [48], intra-axonal water fraction [48], cellularity [95], vascularity and cell size [96], myelin density [10], per-axon diffusion coefficients [97] and others. Several of these models are clinically practicable, and a more detailed comparison is a potential topic for a more specialized work.

However, notes of caution have been raised regarding the (over)simplification of compartmentalizing the tissue by modelling the diffusion [54]. Also, see Burcaw et al [98] and Fieremans et al [99] regarding the impossibility of mapping inner diameters on today’s clinical scanners in humans. Furthermore, we note that, due to unavoidable assumptions and simplifications, even so-called microstructural parameters do not allow us to unambiguously deduce from the MRI data alone which exact differences in tissue microstructure led to the observed differences in diffusion. The NODDI model used in our study is no exception to this. In particular, in order to infer the reported parameters, NODDI assumes fixed values for the diffusivities in its three compartments. This means that values of the ICVF parameter might be biased estimates of the true measurable intra-cellular water fraction, and differences in ICVF might, in part, reflect the model’s inability to adapt to regional differences in diffusivities [52,100]. However, the main goal of our current work is to empirically identify clinically feasible MR-based parameters that allow us to reliably distinguish between regions with known differences in myelin architecture. We found ICVF to be a promising candidate, even though, as for most available alternatives, the mechanism behind it is not fully known.

### Magnetic Resonsance Spectroscopy

Also, MRS parameters (NAA and Cho) were found to be different in frontal compared to the CST region within the centrum semiovale. These regional differences are well documented in the literature [29,30,101], but their interpretation is not straightforward. While NAA is supposed to be a neuronal marker [25,26], Cho is thought to reflect cell membrane turnover [27,28]. Therefore, higher NAA and lower Choline in CST-CS might indicate a higher ratio of glial to axonal cell compartments in the frontal lobe. On the other hand, early studies have shown the involvement of NAA in lipid biosynthesis [102], and may therefore be indicative of a reduced availability due to on-going myelination in frontal areas, or an increased supply of metabolites in the CST. With regard to preferred measurement methodology, the CSI acquisition can be regarded as a more informative approach due to a greater spatial coverage in less acquisition time, at least at clinical magnetic field strengths where the influence of B1 inhomogeneity and the chemical shift artefact on spectral localization is acceptably low. In addition, we observed a greater precision in terms of less variance for CSI than SV, in line with the greater efficiency of the image-based approach.

### Myelin Water Fraction

CPMG based imaging of the myelin water fraction is of interest since it correlates well with histology and yields a parameter which remains unaffected by inflammation or unspecific changes in the overall water content [20,21]. We found this parameter to be increased in the CST area compared to the more frontal regions of the WM. This confirms previous results from MWF measurements of the CST [63]. Whether this is indeed due to increased myelin content, leading to a decreased exchange of water between the extra and intracellular compartments in presence of thicker myelin sheaths, remains to be elucidated. A limitation of our study is posed by the use of the 2D CPMG approach, which did not allow whole brain coverage. Therefore we have no data about the CST located within the PLIC. Nevertheless, this approach allowed us to obtain robust measurements within a relatively short acquisition time (~4min) in a slice which could be matched to the slice used for MRS. Indeed, the draw-back of whole brain, multi-spin-echo based observations of the short T2 component is the long measurement times of 15min or more. This has triggered the quest for alternative, faster, gradient echo-based methods, albeit at the cost of more complex data analysis [103]. This approach assesses the myelin water fraction by constraining fits of the measured MRI data to a three-compartment model of the tissue, including magnetization exchange between the different water pools. The potential bias introduced by the modelling per se thus somewhat hampers this approach. Indeed, it has to be shown to yield MWF values that are several times greater than those furnished by the classical CPMG approach [104].

### Magnetization Transfer Ratio

MTR was found to be increased in PLIC and not different between frontal WM and the CST region located within the centrum semiovale. A previous study also showed higher MTR in PLIC compared to CST in the centrum semiovale [65]. Recently, it was shown that hypomyelinating disorders are accompanied by a pattern of MTR, that closely match histopathological findings with less MT-effects in areas lacking myelin, and increased MTR in areas with more myelin [4,17]. Nevertheless, MTR remains a relative measure, and depends on the exact implementation of the MT-pulses and how these play out in presence of B1 inhomogeneities related to the design of the transmit RF-coil, besides the MR sequence parameters (TR) and the magnetic field strength per se [105–107]. These factors hence need particular attention. In this respect recent advances based on the balanced SSFP sequence hold promise. For instance this approach has been shown to be a robust, sensitive and very fast measure of MTR which generates values that are comparable across multiple measurement sites [7,18,19]. In the present study, we therefore chose to include the fast balanced SSFP MT method as an integral part of our white matter protocol.

### Quantitative Susceptibility Mapping and R2*

The QSM maps showed results that were similar to previous studies [108,109], with very high diamagnetic values in the PLIC, caused potentially by tightly packed and myelinated tracts leading to some form of anisotropy of susceptibility. Indeed, the residual orientation dependence of the major white matter fibre tracts in QSM images is puzzling. Most likely, these variations originate in a myelin-driven anisotropy of the susceptibility, with a greater (diamagnetic) susceptibility variation across the myelinated axons than along them [110–112]. Such phenomena make the QSM calculations more intricate, and may necessitate the introduction of a susceptibility tensor [113][112].and corrections based on the Generalized Lorentzian Tensor Approach (GLTA) rather than the widely used sphere of Lorentz concept [112]. These recent developments and biophysical insights call for the development of validated software tools for the scientific community in the near future. Although we have not developed and used such tools for our data, we did look into the possibility that the obtained data show orientation dependence. For this purpose we investigated the dependence of the frequency maps on the fibre orientation obtained from the DTI data. According to the GLTA theory, cylindrical cavities should not perturb the field when oriented along it, while in case of a perpendicular orientation additional corrections are called for. In view of this, we investigated the voxel-wise variation of the frequency shift maps against the square of the sine of the angle between the external field and the orientation of the diffusion vector. In our limited set of subjects we found significant variation of the frequency shift with fibre orientation consistent with the GLTA in three out of five subjects (data not shown). This kind of analysis could not explain the high QSM values observed in the PLIC area, and therefore other explanations in terms of the g-ratio, fibre density or averaging of effects in fibres with different orientation residing inside the voxels on the susceptibility must be investigated in the future. Such approaches, although more exact, would likely require that images are acquired for different head orientations, and are not (yet) applicable in the clinic, especially if additional MRI modalities are to be acquired. The simpler approach is based on MRI images acquired for a single head orientation, and the final results rely on the selected method for quantification. Several different flavours for QSM quantification based on the sphere of Lorentz approach have been proposed, and these differ mainly by how unwanted background signals are dealt with and which type of mathematical constraints are put on the solution to the inversion problem [114–118].

In contrast to the QSM data, we did find significant orientation dependent effects of R2*, that varied as the fourth power of the sine of the angle between the main DTI orientation and the external field, in accordance with previous reports [111,119,120]. Again the PLIC area stood out in terms of greater than expected values, suggesting consistent effects of a thicker myelin sheet across MRI modalities (QSM, MTR and R2*). On the other hand, in ANT and CST that have similar characteristics in terms of crossing fibers, no significant differences in R2* were found. In future studies, more refined models that incorporate a greater number of microcompartments that give rise to multi-exponential decay may be required[121].

### Towards combining quantitative sequences for the assessment of WM pathology

This study shows that different quantitative MRI parameters might add complementary information for the assessment of WM microstructure. Depending on the underlying tissue architecture of the region of interest, the research question and the suspected type and distribution of WM pathology, a combination of WM MRI parameters might be optimal for the quantification and characterisation of WM microstructure [12].

Furthermore, when studying WM pathology, it is important to have appropriate control data, as MR parameters vary throughout the brain. Not only the cortico-spinal tract [63–65] as demonstrated in our study, but also callosal fibres [11] and other major fibre bundles in the brain have been shown by MR parameters to vary in their microstructural properties [10,122], confirmed by the findings from anatomical ex-vivo histology studies in humans [60,123]. Several of the MR parameters could be validated with histopathology [10,21,124,125].

Moving towards in vivo histology, studies attempt to assess axon diameter, myelin content or measure the g-ratio [10,11,122], which is the ratio between axon and total fibre diameter [126].Advances in this field of MRI based in vivo histology are promising [12]. For example, over the years several studies have shown that even intra-cortical myelin can be probed through the T1-shortening caused by myelin, giving rise to MR signal differences along the cortical rim [127–129]. In white matter fibres, T1 undergoes profound changes in development [130]. Recently, it was shown in adults that variations in T1 can be explained in terms of a combination of other quantitative MR parameters, regardless whether grey or white matter areas are considered [14]. The main contributor to T1 is the magnetization transfer effect, MT, followed by water content and iron, which was assessed in terms of the effective transverse relaxation rate. Therefore, we arguably chose to directly assess these MR parameters, rather than measure T1 in white matter tracts. Since a substantial gain in sensitivity towards microstructure can be obtained by separating the short from the long T1 components [131,132] future studies may prove that this parameter is actually a sensitive, if not specific, measure for assessing myelin.

A critical and limiting factor for MRI protocols used in a clinical setting is the total duration of the exam. Often, trade-offs are made in terms of lower spatial resolution, especially when acquiring a combination of sequences or when subjects do not tolerate long scan times due to their age or brain dysfunction. Choosing carefully the best combination of several sequences allowing a variety of MRI parameters to be analysed [8], and new hardware [133] or acquisition strategies [134,135] will certainly help to overcome this current limitation.

## Conclusion

For the first time, a large number of modern microstructural MRI sequences have been systematically compared on a single well-defined cohort. Within a clinically feasible scan time we were able to assess and characterise WM microstructure using a combination of microstructural MRI sequences. By assessing new quantitative WM parameters we were able to provide normative data and discuss their interpretation in regions with different myelin architecture. The results demonstrate that the underlying normal WM fibre architecture influences significantly and differently the behaviour of several quantitative MRI parameters. In tightly packed parallel fibre bundles high values were observed in all myelin-sensitive parameters, including MTR, QSM, FA, and ICVF, and lower MD and ODI, reflecting more diffusion restriction and less free extracellular water diffusion due to aligned and tightly packed (myelin) membranes. On the other hand, in the frontal WM with underlying crossing fibres architecture, higher MD and Cho, and lower FA, NAA, ICVF, and MWF were observed compared to the more parietal WM, where CST fibres with larger axon diameters and thicker myelin sheaths pass through. These normative data are essential when using MR parameters as biomarker for WM disorders.
